# A Bird’s Eye View of Various Cell-Based Biomimetic Nanomedicines for the Treatment of Arthritis

**DOI:** 10.3390/pharmaceutics15041150

**Published:** 2023-04-05

**Authors:** Nupur Vasdev, Bhakti Pawar, Tanisha Gupta, Mahi Mhatre, Rakesh Kumar Tekade

**Affiliations:** National Institute of Pharmaceutical Education and Research (NIPER), Ahmedabad, Department of Pharmaceuticals, Ministry of Chemicals and Fertilizers, Opposite Air Force Station, Palaj, Gandhinagar 382355, Gujarat, India

**Keywords:** biomimetics, arthritis, bioinspired, nanoparticles, stimuli-responsive, membrane-coated

## Abstract

Arthritis is the inflammation and tenderness of the joints because of some metabolic, infectious, or constitutional reasons. Existing arthritis treatments help in controlling the arthritic flares, but more advancement is required to cure arthritis meticulously. Biomimetic nanomedicine represents an exceptional biocompatible treatment to cure arthritis by minimizing the toxic effect and eliminating the boundaries of current therapeutics. Various intracellular and extracellular pathways can be targeted by mimicking the surface, shape, or movement of the biological system to form a bioinspired or biomimetic drug delivery system. Different cell-membrane-coated biomimetic systems, and extracellular-vesicle-based and platelets-based biomimetic systems represent an emerging and efficient class of therapeutics to treat arthritis. The cell membrane from various cells such as RBC, platelets, macrophage cells, and NK cells is isolated and utilized to mimic the biological environment. Extracellular vesicles isolated from arthritis patients can be used as diagnostic tools, and plasma or MSCs-derived extracellular vesicles can be used as a therapeutic target for arthritis. Biomimetic systems guide the nanomedicines to the targeted site by hiding them from the surveillance of the immune system. Nanomedicines can be functionalized using targeted ligand and stimuli-responsive systems to reinforce their efficacy and minimize off-target effects. This review expounds on various biomimetic systems and their functionalization for the therapeutic targets of arthritis treatment, and discusses the challenges for the clinical translation of the biomimetic system.

## 1. Introduction

There are more than a hundred different kinds of arthritis, but osteoarthritis (OA), rheumatoid arthritis (RA), inflammatory arthritis, and psoriatic arthritis are the most frequent. Inflammation, monocyte infiltration, joint stiffness, synovial swelling, pannus development, and articular cartilage degradation are universal features of all forms of arthritis. It was often believed that articular cartilage loss and inflammation in a joint resulted from chronic loading and poor biomechanics in the joint. Following this, the affected individual had pain, oedema, and inability to move freely. Current understanding has expanded OA’s complexity to include metabolic and inflammatory components in its process [1].

Inflammatory arthritis is caused by autoimmune inflammation that damages joint tissue. Despite many investigations, a clear reason has not been found. It is most likely that this happens when an individual with a genetic predisposition is exposed to a certain environmental trigger. RA ranks as the most frequent form of inflammatory arthritis. Reactive arthritis, psoriatic arthritis, and ankylosing spondylitis are examples of spondyloarthropathies, which are slightly less prevalent than other forms of arthritis [2]. Clinical indications of psoriatic arthritis vary since the disease is heterogeneous and complex. Individuals may have musculoskeletal indications, such as axial or peripheral arthritis dactylitis, enthesitis, and tendinitis, but non-musculoskeletal involvement, such as nails, eyes, and stomach, is also frequent, in addition to manifested or latent psoriasis. Individuals with psoriatic arthritis are also more likely to develop psychological, cardiovascular, and metabolic complications [3]. However, the specific cause of arthritis is still uncertain, and the disease has no permanent cure. The patient’s function can be restored with surgical therapy, and the symptoms or disease condition can be managed with medication. Yet, thus far, no successful therapy approach has addressed the underlying aetiology of arthritis [4]. Although anti-inflammatory medicines are widely used to treat arthritis, they can show severe adverse effects, including a high risk of cardiovascular disorders, heart attacks, and gastrointestinal bleeding. More prognostic indicators and innovative treatment options are desperately needed for these individuals [5,6,7].

RA can be treated using disease-modifying anti-rheumatic medications (DMARDs). These can alleviate the severity of RA, improve function, and slow the disease’s progression. Corticosteroids, analgesics, and non-steroidal anti-inflammatory drugs (NSAIDs) are all useful in relieving symptoms and are often used as part of adjuvant treatment with DMARDs. Hence, all existing therapies help in controlling the arthritic flares for symptomatic relief. There is need for the advancement of curative treatment modalities [4].

With a 95% confidence range, the predicted prevalence of RA worldwide is 0.46% (0.06–1.27). Between 1986 and 2014, the point prevalence of RA was 0.45% (95% CI 0.38–0.53%), and between 1955 and 2015, the pooled period prevalence of RA was 0.46%. Between 1980 and 2019, the average annual rate of new cases of RA per 100,000 people was 460, albeit this number varied by region and research design [8]. Many health factors are affected by the growing prevalence of OA across the world. Obesity and joint damage are strongly associated with an increased risk of OA; thus, it is imperative that people make more effort to reduce these risks in healthcare settings. Approximately 240 million people have symptomatic OA, with 10% of males and 18% of women over 60 being affected [9].

One of the numerous benefits of nanotechnology is the ability to target and treat certain medical situations with extreme precision. In response to this, scientists are turning to nanotechnology as a viable source of innovative arthritis treatments. Nanocarriers have been demonstrated to improve medication solubility, circulation time, and reduced clearance, and target therapeutic delivery to targeted areas in a controlled manner [10]. Drugs can be passively or actively delivered to specific cells, tissues, or subcellular domains using medicine and nanotechnology combination in the form of nanomedicines [11]. Because of the engineering possibilities of nanocarriers, the bioavailability of these bioactive compounds can be increased by preventing degradation. As chronic inflammatory conditions and RA involve widespread systemic inflammation, enhanced permeability and retention (EPR) is the most critical consideration for RA treatment. Increasing the circulation time of nanoparticles (10–1000 nm) by surface functionalization with effective functional groups can assist in maximising their value in executing their intended purpose. EPR allow the nanomedicine to concentrate on the affected joints by passive targeting [12].

Biomimetic nanomedicines have characteristics mimicking the biological system. Potentially useful solutions for developing disease-specific nanoplatforms can be found in biomimetic nanotechnology. In subsequent biomedical studies, the membrane-engineered nanoplatforms created by coating various cellular-system-based membranes onto synthesized nanoparticulate cores, which inherit antigenic characteristics from source cell lines, perform as source cell lines maintaining their full membranous functionalities [13]. Compared to traditional nanocarriers, biomimetic nanocarriers are preferred due to their increased specificity in transporting the therapeutic component to the desired location, which has several applications in clinical diagnostics.

Nanoparticles have been reported to permeate into the arthritic joint cavity through passive targeting, resulting in improved transportation and therapeutic efficacy as compared to free therapeutics with less systemic toxicity and adverse effects [10]. They are further distinguished by the fact that they are biodegradable in nature. The preparation and evaluation of leflunomide-loaded poly(-caprolactone) nanoparticles as local drug carriers leads them to being capable of reducing inflammation associated with RA. Intra-articular nanoparticles may be a possible therapeutic option to systemically deliver medicines for arthritis treatment without causing systemic side effects [14]. A nanostructured lipid-carrier-based smart gel of methotrexate was formulated with a prolong localised effect, biocompatibility, and being non-toxic to synovium to treat rheumatoid arthritis [15]. A diclofenac-diethylamine-loaded microsponges-based gel was formulated to allow prolonged drug release for arthritis treatment [16].

Additionally, mammalian-derived biomimetic nanocarriers aid in preventing an immunological reaction [17]. Because of their success in imitating the properties of the biological system, biomimetic nanocarriers have seen extensive advancement; one highly promising class of them is cell-derived biomimetic carriers. Nanocarriers generated from cell-derived membranes surrounding payloads may inherit the properties of the parent cells, including lengthy circulation, immune escape, and recognition ability, due to the diversity of carbohydrates and proteins on cell membranes [18]. Plasma proteins readily opsonize bare-surfaced nanomedicines, facilitating their removal by the mononuclear phagocyte system (MPS). In addition, the effectiveness of nanoparticles is greatly diminished because of their propensity to passively accumulate in inflamed joints and then release medicines into each type of cell in the articular cavity [19,20]. Therefore, functionalization of the nanomedicines is required to minimize these effects and enhance the efficiency of the therapeutic agent.

Components and biomolecules derived from cellular membranes are used to functionalize biomimetic nanomedicine, improving its biocompatibility, target specificity, and stability. Numerous cell sources such as platelets, RBCs, lymphocytes, cancer cells, bacterial cells, and mesenchymal stem cells can be used to biofunctionalise the nanomedicines. These biomimetic nanomedicines can be used for targeted drug delivery with the help of ligands composed of antibodies, aptamers, small molecules, and peptides. Stimuli such as pH changes, magnetic fields, light, heat, oxygen, and redox reactions are all used in active therapies using stimuli-responsive biomimetic nanomedicines. However, the fragile nature of the membrane and orientation of the membrane with nanomedicine are some of the challenges that researchers face in the clinical translation of biomimetic nanomedicines. In this review, we summarize various targets for arthritis, cellular-system-based biomimetic nanomedicines, their functionalization with a targeted ligand or stimuli-responsive systems for arthritis treatment, and the challenges to conquer for clinical translation of these biomimetic nanomedicines. Currently available reports of biomimetic nanomedicines for the treatment of arthritis talk broadly about the biomimetic system being similar to a virus-based, bacteria-based, biomolecules- and cell-based system. Here, we focused particularly on the cellular-system-based biomimetic system available for arthritis treatment, and the preparation of those cellular-based biomimetic systems, their limitations and applications are also explored.

## 2. Pathophysiology and Therapeutic Targets of Arthritis

RA is a long-lasting autoimmune disorder that causes inflammation in the synovial membranes and the degradation of bone and cartilage. One percent of the population suffers from RA, with women being three times more likely to be diagnosed than males. Even though scientists worldwide are still investigating the specific cause of RA, the illness is thought to have several causes, including environmental and genetic factors [21]. Recently arising protein targets that significantly impact the adaptive and innate immune response in RA include interleukin-1 receptor-associated kinase (IRAK)-4, interleukin (IL)-4, IL-15, IL-10, IL-18, IL-17, and IL-23. Prostaglandins (PGs), platelet-activating factor (PAF), lipoxins (LXs), nitric oxide (NO), leukotrienes (LTs), and reactive oxygen species (ROS) are small molecular metabolites that play important roles in the RA pathophysiology. Moreover, a growing body of evidence suggests that epigenetic regulators, such as RNA methylation, DNA methylation, non-coding RNAs, and histone modifications, play critical roles in RA [22]. During RA, both memory and naïve B cells infiltrate and concentrate in synovial tissue, presumably experiencing persistent activation of specific B cell clones with strong migratory ability [23].

The proliferation of inflammatory cells and FLSs uses up a lot of oxygen, leading to low pH and hypoxia in the microenvironment of rheumatoid arthritis, which in turn triggers the angiogenesis, generation of reactive oxygen species (ROS), and infiltration of inflammatory cells. Similarly, inflammatory stimuli can increase the concentration of reactive oxygen and nitrogen species (RONS) in the arthritis microenvironment, and vice versa. A distinctive vicious loop involving many inflammatory factors and multiple RONS serves a crucial role in the development of RA. The chemical and physical characteristics of several polymer-based nanostructures can be altered by reacting with RONS. Various RONS responsive nanocarriers, nanozymes, and carbon nanotubes scavenge the RONS and assist in the RA as well as other inflammation treatment [24,25].

**Figure 1:**
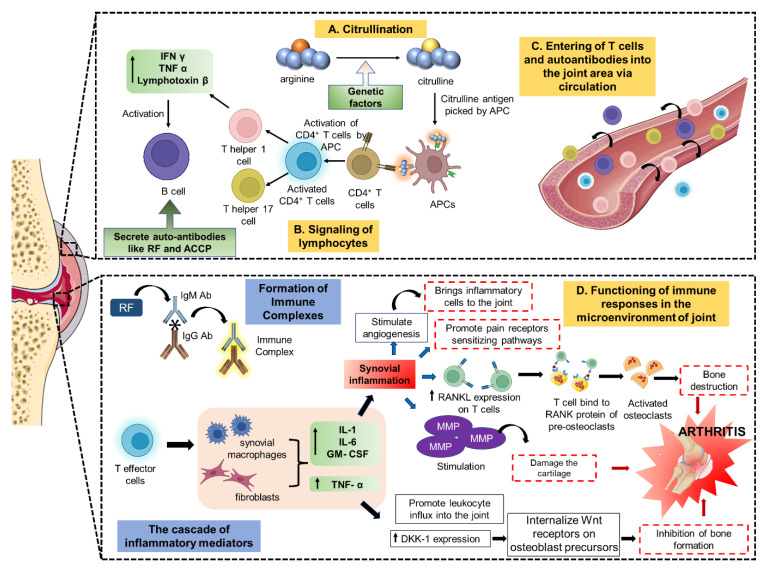
Pathophysiology of Rheumatoid Arthritis. (**A**) The immune system interprets citrulline-containing regions of a number of proteins as foreign antigens, and the APCs subsequently take these antigens up. (**B**) The APCs are transported to the lymph nodes, where the T cells are stimulated and develop into B cells and T helper cells. (**C**) The lymphocytes go through the circulatory system to the joint region. (**D**) In the meantime, the T effector cells release pro-inflammatory mediators that, through a cascade of events of other inflammatory mediators, cause joint inflammation, an increase in pain receptors, and bone loss. All these events eventually cause the development of arthritis. APC: antigen-presenting cell; IFN: interferon; TNF: tumour necrosis factor; RF: rheumatoid factor; ACCP: anti-cyclic citrullinated peptides; GM-CSF: granulocyte–macrophage colony-stimulating factor; RANKL: receptor activator for nuclear factor κ B ligand; MMP: matrix metalloproteinases.

The synovial joint’s cartilage is made up of chondrocytes, and chondrocytes synthesize an extracellular matrix (ECM) rich in glycosaminoglycans (GAGs) and type II collagen. As a result of targeted adhesion and invasion, the hyperplastic synovium is primarily responsible for the significant deterioration of the cartilage in RA. RA is characterized pathologically by a loss of bone, which can either be localized, periarticular, or even systemic. Inducing osteoclasts and inhibiting osteoblasts leads to bone resorption. Osteoclast differentiation and the development of inflammatory infiltrates in the subchondral bone marrow are believed to be responsible for “periarticular” bone loss [26]. In RA, inflammation starts in the synovium, and the lining of the synovium is the primary source of inflammatory proteases and cytokines, which work with activated osteoclasts and chondrocytes to cause joint destruction. Signalling pathways of chemokine- and cytokine-mediated inflammation, the p53 pathway, angiogenesis, T-cell activation, de novo purine biosynthesis, the vascular endothelial growth factor (VEGF) receptor signalling pathway, and the apoptosis signalling pathway are some of the most commonly used targeted pathways in prevailing RA treatment. Consequently, non-steroidal anti-inflammatory medications (NSAIDs), disease-modifying anti-rheumatic medicines (DMARDs), and corticosteroids are the major therapeutic treatments used to treat RA [27]. The pathophysiology of rheumatoid arthritis is shown in Figure 1.

### 2.1. Intracellular Targets

Intracellular signal transduction pathways allow cells to react and adjust to environmental stress. In most cases, alterations in protein expression or gene transcription result from activating a signalling cascade initiated by ligating certain cell membrane receptors that perceive the external environment. These pathways are activated in response to a pro-inflammatory stimulus, which can have beneficial or detrimental effects on the host depending on the context (i.e., whether the reaction is to an acute infection or an ongoing autoimmune illness such as RA). Studies analysing the signal transduction pathways have revealed possible treatment targets in addition to improving our understanding of the aetiology of RA [28].

Intracellular signalling is transduced by the transcription factor STAT, which is regulated by the tyrosine kinase JAK. The JAK-STAT cascade is the primary signalling pathway for many growth factors and human cytokines. When ligand binding causes receptor subunit crosslinking, JAK is activated intracellularly. Activated JAKs phosphorylate the receptor’s tyrosine residues to facilitate STAT binding in the SRC2 homology domain. This tyrosine phosphorylation by JAK causes STATs’ activation and dimerization. After activation, STATs translocate from the cytoplasm into the nucleus, activating the transcription of certain genes [29].

There are four members of the JAK family: JAK1, JAK2, JAK3, and Tyk2. However, only a subset of haematopoietic cells expresses JAK3, while JAK1, JAK2, and Tyk2 are widely expressed. Many different cytokines can interact with JAK1 via the common chain receptor subunit (IL-2 and IL-4 receptor family) and the glycoprotein-130 subunit. Similarly, JAK1 is engaged in the signal transduction for type 1 interferon. Through the glycoprotein-130 subunits, JAK2 communicates with cytokines and a wide variety of other hormones such as thrombopoietin, erythropoietin, growth hormone, and prolactin. Therefore, there may be a link between thrombocytopenia and anaemia and the usage of JAK2 inhibitors. Researchers have investigated several different JAK inhibitors for RA treatment [30,31].

Phosphoinositide 3-kinases (PI3Ks) are enzymes involved in lipid signalling; they catalyse the formation of phosphoinositide and inositol phosphate lipids. Structure, regulation, and the types of lipid substrates that each class of PI3K prefers have led to the classification of PI3Ks into three distinct classes. Class I PI3Ks regulate a wide variety of biological processes, including cell cycle progression, survival, proliferation, and apoptosis, as well as the adhesion and migration of leukocytes. PI3Kγ and PI3Kδ are being studied as potential pharmaceutical targets for the treatment of rheumatoid arthritis. Mice given a p110γ-specific inhibitor have much less joint inflammation in the CIA model. Neutrophil infiltration and synovial inflammation in arthritic joints are dramatically reduced histologically as well. A lack of p110γ in synovial fibroblasts results in less severe TNF-mediated cartilage degradation and inflammatory-erosive arthritis by reducing the production of matrix metalloproteinases in chondrocytes and fibroblasts [32,33,34].

Various other signaling pathways involved in the RA are MAPK signaling, Wnt signaling, the notch signaling pathway, and the NF-κB signaling pathway. Cascade protein kinases RAF, RAS, extracellular signal-regulated kinases (ERK), and mitogen-activated protein/extracellular signal-regulated kinase (MEK) are all components of the mitogen-activated protein kinase (MAPK) pathway. Hormones, neurotransmitters, physiological stress, inflammatory substances, growth factors, and viruses are some of the extracellular stimuli signals that might activate the MAPK signalling pathway. The MAPK signalling system regulates cell differentiation, proliferation, and cell death by triggering a variety of cellular responses. There are three primary subfamilies of MAPKs; c-Jun N-terminal kinase (JNK), ERK, and p38 MAPK. Each MAPK signal activation is governed by a three-tiered kinase module [35]. The wnt signaling pathway involves several processes such as cell renewal, bone resorption, bone formation, organ development, and tumorigenesis. Non-canonical (beta-catenin-independent) pathways and canonical (beta-catenin dependent) are the two primary wnt signaling pathways. Patients with RA have increased expression of genes such as Fz and Wnt in the synovial membrane. In glycoproteins, the Wnts bind to Fz family receptors on the surface of cells and the Wnt/Fz complex regulates the formation of tissue throughout joint formation, limb development, and embryogenesis. By promoting osteophyte formation, Wnt signalling activation may be linked to the anabolic model in joint remodelling seen in ankylosing spondylitis patients, while blocking Wnt signalling may contribute to the catabolic model in bone remodelling in RA patients by promoting bone erosion. When the Wnt/beta-catenin signalling pathway is activated in chondrocytes, cartilage matrix breakdown ensues, analogous to what happens in OA and RA [36]. Multifaceted activities in both adaptive and innate immune cells are controlled by the nuclear factor kappa B (NF-B) signalling pathway. The NF-kappa B transcription factor is involved in cell survival, differentiation, and proliferation. Over the course of the illness, fibroblast-like synoviocytes (FLSs) undergo alterations that cause them to release inflammatory mediators such as matrix metalloproteinases and inflammatory cytokines that erode the cartilage and joints. The inflammatory characteristics of RA have been linked to aberrant NF-kappaB activation. In addition to promoting RAFLS proliferation, NF-B activation can also prevent FLS apoptosis, leading to hyperplasia in the synovium of RA [37]. A group of highly conserved cell-surface receptors called “Notch” genes control cell growth in species as diverse as sea urchins and humans. Many aspects of normal cell development are influenced by Notch signalling. These aspects include pluripotent progenitor differentiation, cell division, apoptosis, and the establishment of cell borders. Macrophages, synoviocytes, and synoviocytes with a fibroblast-like phenotype are all stimulated to produce pro-inflammatory cytokines when Notch signalling is expressed and activated to worsen RA. The Th17 differentiation process is controlled by Notch-1 binding to the ROR-γT promoters and IL-17. Blocking Notch-3 reduces the activation of Th1 and Th17 cells in CIA mice, suggesting a function for Notch-3 in the development of antigen-specific T-cell development. Moreover, Notch-3 was discovered to be significantly increased in synovial fibroblasts, and in a mouse model, and inhibiting Notch-3 signalling decreased inflammation and protected against joint damage [38]. Another essential intracellular kinase being studied for treating RA and other immune-mediated illnesses, including SLE, is Bruton’s tyrosine kinase (BTK). Expression of this non-receptor tyrosine kinase is confined mainly to B cells and myeloid cells such as dendritic cells and macrophages. It is essential for the growth and activation of B cells [32]. Various therapeutic targets for arthritis treatment are listed in Table 1.

TNF, tumour necrosis factor; IL-1R, IL-1β, IL-6R, IL-6^a^, IL-2, IL-10, IL-15, IL-17, IL-17R, IL-18, IL-23, interleukin (IL)-1 receptor, -1 beta, -6 receptor, -6 antibody, -2, -10, -15, -17, -17 receptor, -18, -23, respectively; TGF-β, transforming growth factor-beta; IFN-γ, interferon-gamma; GM-CSF, granulocyte–macrophage colony-stimulating factor; GM-CSFR, granulocyte–macrophage colony-stimulating factor receptor; Ab, antibody; JAK, Janus kinase; IRAK-4, interleukin (IL)-1 receptor-associated kinase 4; p38 MAPK, mitogen-activated protein kinases; MMP-9, matrix metalloproteinase 9; CD20, CD80, CD3, CD11a, CD19, cluster of differentiation (CD)-20, -80, -3, -11a, -19, respectively; GRK2, G protein-coupled receptor kinase 2; BMP9, bone morphogenetic protein 9; TLR4, toll-like receptor 4; MEK, mitogen-activated protein kinase; BTK, Bruton’s tyrosine kinase; CXCL10, CXCL12, CXCL13, CXCL16, CXC motif ligand-10, -12, -13, -16; CXCR1/2, CXCR3, CXCR4, CXCR7, CXC motif receptor-1/2, -3, -4, -7; CCL2, CC motif ligand 2; CCR1, CCR2, CCR5, CCR7, CCR9, CC motif receptor-1, -2, -5, -7, -9; CX3CL1, CX3C ligand 1. Adapted from [22] with slight modifications; licensed under a Creative Common Attribution 4.0 Generic License (https://creativecommons.org/licenses/by/4.0/), accessed on 11 March 2023.

### 2.2. Extracellular Targets

Multiple extracellular targets are currently under investigation. The majority involve earlier targeted cytokines, such as IL-6 ligands or IL-6R. Recent research on various cytokines, such as IL-21, IL-20, and IL-17, has yielded unsatisfactory results. Myeloid cells, such as dendritic cells, neutrophils, and macrophages, are differentiated and proliferated in response to granulocyte–macrophage colony-stimulating factor (GM-CSF), a haematopoietic growth factor. Endothelial cell migration and proliferation are also stimulated by GM-CSF. Multiple kinds of cells, including lymphocytes, myeloid cells, and tissue-resident cells such as osteoblasts, chondrocytes, endothelial cells, and fibroblasts, all contribute to its production [32].

GM-CSF mediates a significant part of the innate immune response. The generation of inflammatory cytokines, overexpression of adhesion molecules, and stimulation of phagocytosis are all typical consequences of this enhancement of the effector actions of neutrophils and macrophages. GM-CSF can polarise macrophages engaged in the inflammation of the synovial membrane into an inflammatory M1 phenotype [39].

The synovial membrane expresses GM-CSF, and elevated levels of GM-CSF were observed in the synovial fluid of RA patients. GM-CSF receptors are overexpressed on circulating monocytes, which encourages maturation and macrophage activation in the synovium. Moreover, synovial tissue showed upregulation of GM-CSF receptors, and in vitro research suggests that GM-CSF stimulates the growth of fibroblast-like synoviocytes. It has been found that GM-CSF aids in the growth and survival of both dendritic cells and Th17 cells. GM-CSF protects monocyte-derived dendritic cells against the immunosuppressive effects of IL-10 in vitro, allowing them to retain their inflammatory capacity. Dendritic cells are involved in the pathophysiology of RA because they can present autoantigens through MHC molecules and generate large amounts of inflammatory cytokines such as TNF-α, IL-1, and IL-6 [32,40,41].

## 3. Biomechanics for Application of Biomimetic Systems in Arthritis

Various approaches are available for the design and development of biomimetic systems in arthritis, such as their shape, surface, and movement

### 3.1. Biomimetic Surface

The development of biomimetic systems is greatly aided by nanotechnology. However, it has some limitations due to the dynamic biological environment. To overcome the limitation, surface modification is performed by attaching ligand moieties or targeting moieties to the surface. By imitating the body’s typical physiology, the immune system of the host could be compromised [42]. To avoid scavenging by the reticuloendothelial system and enhance the circulation time, the stealthy nanoparticle can be prepared. The surface of the nanoparticle can be decorated with PEG-coating. PEGylated nanoparticles show an enhanced circulation time [43]. Less immunogenic and cell-toxic nanoparticles can be created by covering them with stealth polymers. Poly-2-oxazolines (POX) are a newly emerging PEG substitute since anti-PEG antibodies have been identified as a widespread issue, according to a recent study [44]. Nevertheless, because of their immunogenicity and potential to trigger allergic reactions, these stealth coats and several other polymers are ultimately being criticized in terms of their usefulness as a powerful protectant.

### 3.2. Biomimetic Movement

Movement is a fundamental behavioural characteristic of species and a behavioural reaction to changes in the internal and external environment. Living organisms frequently contain materials that can change their mechanical characteristics, which are essential to how living things move. The imitation of biological movement is a crucial task [45]. As a result, significant attempts have been made to emulate such designs and movement behaviors by fully utilizing the characteristics of stimuli-responsive innovative materials to design intelligent materials and machines with particular purposes [46]. Using bio principles to create innovative solutions is known as “bioinspired design”. By thoroughly comprehending how organisms move, researchers can create and actualize biomimetic motion by utilizing the tunable biomechanical capabilities of the material. Usually, biomimetic movement based on micro or nanorobots is widely used in biomedical applications. An example of bacteria-inspired/biomimetic movement is microbots. Microbots are described as nanoparticle delivery systems based on bacteria. For the transport of a drug into the cell, they carry nanoparticles on their surface. Since the drug is already loaded into the nanoparticle’s surface, genetic engineering is unnecessary for drug delivery [47,48].

### 3.3. Shape and Surface

Numerous studies have been conducted to improve the shape of biologically inspired nanoparticles by examining several morphologies, such as the rod, sphere, cubic, star, prism, and disc shapes. In the process of macrophage internalization, nanoparticle geometry is crucial. For instance, spherical nanoparticles are less likely to concentrate in mononuclear phagocyte system (MPS) organs than discoidal nanoparticles, which accumulate in the lungs and spleen [49]. The shape of biomimetic nanoparticles must be taken into consideration since different morphologies can trigger various intracellular signalling pathways. Depending on each one’s unique shape, they can, for instance, encourage the secretion of numerous cytokines [50]. In order to effectively comprehend the biological effects of biomimetic nanoparticles, it is vital to analyse their shape. Typically, rigid, spherical nanostructures are used. By imitating the texture and structure of the cell, it is possible to improve the functionality and targeting of the cell. Researchers have been successful in creating a novel type of hollow-structured mesoporous carbon nanocapsules (HMCNs) with nano-size monodispersity and uniformity, hollow core/mesoporous shell nanostructures, hydrophilicity, and most notably, red blood cell (RBC) morphology, making the material a very encouraging nanoplatform for smart drug delivery/releasing, metastasis inhibition, and MDR reversal targeting, the three most significant challenges of metastasis, drug resistance by cancer cells, and the side effects of chemo drugs in cancer treatment [51].

The release of a therapeutic moiety at the right targeted site is essential. It can be attained by modifying or decorating the surface of a nanostructure so that it will recognize or respond to the targeted site environment and release the therapeutics [52]. Moreover, the release of the therapeutics can be manipulated using different stimuli such as a magnetic field, ultrasound, and light [53]. A peptide with self-assembling properties can be used to create a biomimetic system quickly and efficiently. These self-assembled peptides are amphiphilic; hydrophobic parts interact with similar molecules, creating a wide structure, and on the other hand, hydrophilic structures interact with the body fluids [54]. This self-assembling nature of lipids helps to prepare biomimetic systems. In addition, the lipid bilayer mimics the cell membrane, thereby promoting the transfer of substances from the core of the nanoparticles. The release of the therapeutic moiety can be triggered in various ways; for example, the integration of the ion channel protein to the lipid bilayer allows adjustable drug release [55]. Polymers that are synthetic can also simulate channel functioning. Thus, stimuli-responsive or smart drug delivery systems can benefit from the surrounding environment of the targeted site. In the study of drug permeability and regenerative medicine, biomimetic membranes have attracted a lot of attention. Mucus has a critical role in affecting drug penetration and absorption, although in vitro models designed to replicate epithelial surfaces frequently ignore this. Hence, in the current scenario, researchers are focusing majorly on the muco-mimetic systems. Mucus inclusion in these models should enhance the IVIVC and, ideally, minimise the need for preclinical testing [56]. An example of a biomimetic functional system is a microgel that releases the medication in response to temperature or concentration [57].

## 4. Cell-Membrane-Coated Biomimetic Nanomedicine for Arthritis

A cell-membrane-coated biomimetics system combines an isolated cell membrane with a template of choice to imitate the function of a cell. These mimics are biocompatible, improve bio-interfacing skills, modify the physicochemical properties, and protect the encapsulated drug by avoiding degradation [58]. A cell membrane with a diameter of 5–10 nm often consists of various lipids, carbohydrates, and proteins, each having their essential roles as lipids are accountable for the rigidity and integrity of the cell membrane. In contrast, carbohydrates give fluidity, and proteins govern adhesion as well as cell signalling. This cell membrane interacts with the biological environment and carries out intricate biological processes to survive and grow [59]. Different cell types possess different cell membranes, such as RBC cell mem., platelet cell mem., NK cell mem., macrophage cell mem., etc. In the following section, we discuss the procedures for isolating cell membranes from various cell types.

### 4.1. Isolation of Cell Membrane

**Figure 2:**
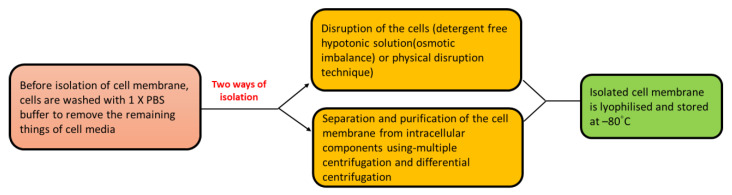
Overview of the isolation of cell membrane.

**Figure 3:**
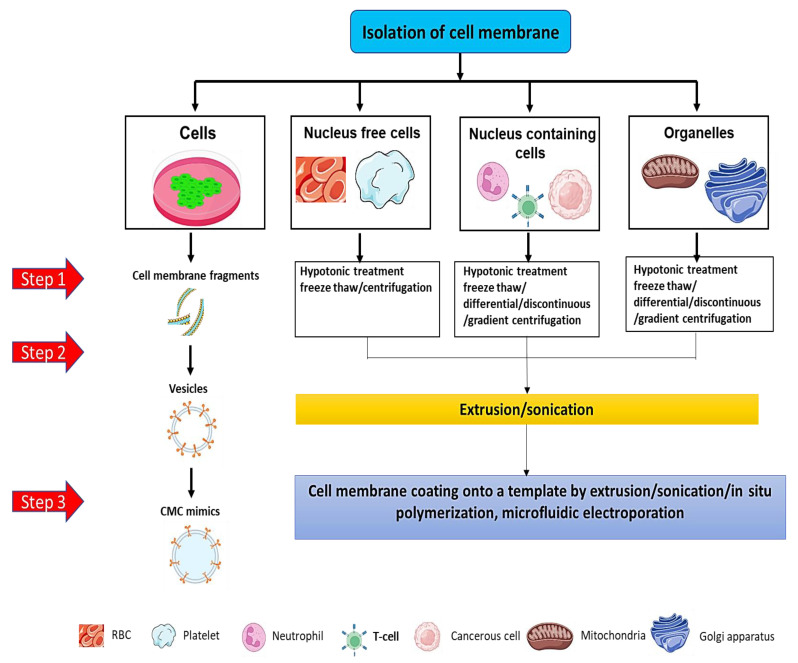
Isolation of the cell membrane from different types of the cells such as nucleus-containing cells, nucleus-free cells, and organelles.

Usually, cells are of two types; nucleus-containing cells and nucleus-free cells. Isolation of the cell membrane involves separating the cell membrane from different cell forms with minimum contamination of the cytosol, mitochondria, and nucleus. The reason for using a pure cell membrane is that it aids in facilitating the formation of cell membrane-coated mimics by improving the effective and homogenous surface coatings with maximum imitation of the functions of the cell on templates. Furthermore, to avert the deterioration of proteins present on the cell membrane, the addition of a combination of protease/phosphatase inhibitors is performed. This process is usually carried out in the extraction buffers with pH 7–7.4 in an ice-cold condition [60,61]. Mainly two types of isolation processes are performed, as shown in Figure 2. In addition, the cell membrane isolation protocol is slightly different from cell to cell, such as nucleus-containing cells, nucleus-free cells, and organelles isolated from the cell-containing nucleus, as shown in Figure 3.

### 4.2. Formulation of Cell-Membrane-Coated Nanomedicines

In the current scenario, nanomedicine has developed a novel drug delivery system that involves the formation of nanoparticles that can be coated with the cell membrane. When a person receives nanoparticles, they come into contact with an extraordinarily complex and occasionally harsh environment designed to detect and eliminate foreign materials [62]. However, in the bloodstream, specific proteins and the reticuloendothelial system (RES) can stop the nanoparticle from approaching its targeted site. To avoid this RES scavenge, nowadays, stealth nanoparticles are synthesized by attaching with polyethylene glycol (PEG), which also helps to improve the stability of the nanoparticles [63]. However, a rise in immunological reactions to synthetic PEG is starting to be seen, which could influence how effective these nanocomposites are. The complex capabilities necessary for proper interaction with biological tissues can be directly replicated on the surfaces of nanoparticles thanks to this innovative method of cell-coated biomimetics. In 2011, the RBC membrane was first used to coat the nanoparticles prepared from the poly-(lactic-co-glycolic acid) [64]. Due to the extended circulation period of RBC, i.e., 120 days, the RBC membrane was selected for the coating of nanoparticles.

Recently, researchers have designed exosome-based biomimetic nanoparticles for rheumatoid arthritis (RA). RA can be effectively treated by glucocorticoids. However, because of their severe side effects due to off-targeting, they have clinically limited value. To overcome these challenges, exosome-based nanoparticles were prepared to treat inflamed joints in RA patients. Dexamethasone sodium phosphate was entrapped inside the biomimetic exosome, and its surface was decorated with the folic acid–polyethylene-glycol–cholesterol to obtain a specific targeting action [65].

Another example of a nanoformulation prepared to treat RA is the anti-RA compound schisanlactone E isolated from Tujia ethnomedicine Xuedong by scientists. This compound was incorporated into Prussian blue (PB) nanoparticles. The further surface coating was performed with an RBC membrane with HA modification on the (PB) nanoparticles. Interestingly, results have shown that the circulation time of nanoparticles increased. Reduced synovial hyperplasia was observed, thus giving better therapeutic efficiency [66].

Li et al. designed a dual targeting approach for the treatment of RA. They prepared the self-assembled triptolide nanoparticles facilitated by dipeptide diphenylalanine. These nanoparticles are then encapsulated inside the mannose-modified RBC membrane. This has been shown to be effective at the specific targeting and suppression of synovial hyperplasia [67].

A versatile strategy to target pro-inflammatory M1 macrophages in active RA lesions was put out by Shi et al. [68]. They coated nanoparticles with a Human Umbilical Vein Endothelial Cells (HUVEC) cell membrane overexpressing a tumour necrosis factor-related apoptosis-inducing ligand using gene engineering techniques (TU-NPs). The TU-NPs not only caused M1 macrophages in RA lesions to kill, but they also blocked the action of cytokines to minimize inflammation, stop pannus development, and control synovial hyperplasia. In one such study, in order to treat RA, Li et al. created a macrophage-derived macrovesicle (MMV) and coated it with tacrolimus-loaded NPs [69]. When delivered intravenously, MMV-tacrolimus NPs outperformed bare tacrolimus-loaded and red-blood-cell-membrane-coated nanoparticles in terms of their ability to target RA lesions. The authors further postulated that the macrophage membrane would engage in a deal for the binding of inflammatory cytokines, suppressing osteoclastogenesis to stop RA-related bone loss [4].

In addition to the abovementioned example, neutrophil-membrane-coated nanoparticles were prepared for the management of RA. By joining the neutrophil membrane to polymeric cores, Zhang et al. created membrane-coated nanoparticles that have been shown to have anti-inflammatory properties, inhibit the release of pro-inflammatory cytokines, provide strong chondroprotection against inflamed joints, and specifically target the deep matrix of cartilage. These membrane-coated nanoparticles show a significant therapeutic effectiveness in the collagen-induced and human transgenic arthritic mouse models by reducing joint damage and arthritic severity [70,71].

Similarly, in order to acquire target ability for treating arthritis, the neutrophil membrane was incorporated into erythrocyte-membrane-coated copper nanoparticles (D-CuS@NR NPs) that were loaded with dexamethasone sodium phosphate (Dexp). The synergistic treatment was obtained using NIR laser irradiation. Due to its coating of the cell membrane and ability to release drugs in response to photothermal heat under NIR irradiation, this biomimetic nanoparticle demonstrated considerable cytocompatibility and anti-inflammatory properties in vitro. By local warming and targeted drug distribution, these new hybrid-membrane-coated CuS NPs demonstrated remarkable therapeutic efficacy during in vivo studies [72].

## 5. Extracellular Vesicles-Based Biomimetic Nanomedicine for Arthritis

**Figure 4:**
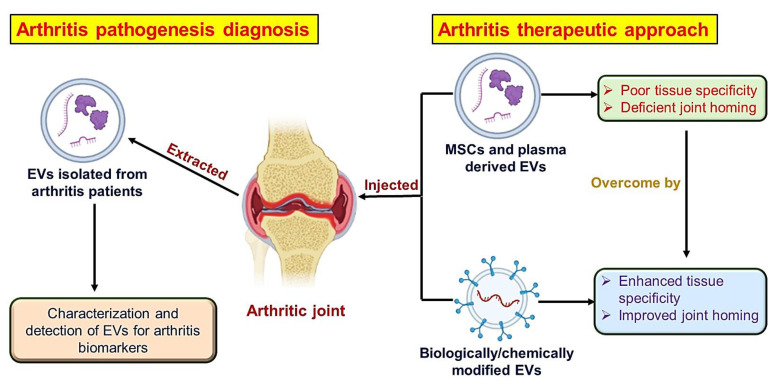
Role of extracellular vesicles in the diagnosis and treatment of arthritis.

Extracellular vesicles (EVs) are small heterogenous membrane vesicles that are enveloped by a bilayer of phospholipid and have 30–5000 nm diameters. They have been secreted by diverse cells that interact with one another through paracrine signaling [73]. Almost all cell types secrete them, and they can be found in various biological fluids, including cerebrospinal fluid, blood, breast milk, urine, saliva, and others [74]. The word EVs is frequently utilized as an overarching term, but it may be further subdivided into apoptotic bodies, microvesicles, and exosomes, depending on their specific sizes. Apoptotic bodies (AB) are dead cell blebs that form during the late phase of apoptosis with a size range of 1000–5000 nm. Microvesicles are vesicles that form after cell stimulation or stress, such as hypoxia or apoptosis, and are available in a size range of 100–1000 nm. Exosomes originate in the endosomal system and are secreted continuously by all types of cells with a size range of 30–150 nm [75,76]. Ultracentrifugation is a common method for isolating EVs from other fluids, such as blood or culture medium used in cell culture. Filtration, affinity-based techniques, size-exclusion chromatography (SEC), and flow field-flow fractionation have all been used to isolate small EVs from larger EV subpopulations (such as apoptotic bodies) [77]. EVs can fuse with target cells’ plasma membranes, be internalized via endocytosis, or communicate with surface receptors on cells to initiate intracellular signalling pathways. When EVs are internalized, they can release proteins, lipids, and nucleic acids such as mRNA and miRNA, which are functional inside cells. EVs may have a pathogenic outcome and hasten disease progression in a pathological setting, but they can also play a protective role and halt the disease’s progress [74,78]. Extracellular vesicles are extensively engaged for the diagnosis of arthritis as well as its treatment (Figure 4).

All types of joint cells secrete EVs, which may play a role in disease progression by promoting inflammation or serving as a pathological signal [79]. The lining cells of synovial, which include synovial macrophages and fibroblasts, are another major EVs source in the joint. Osteoarthritis (OA) articular cartilage EVs range in size from 50 to 250 nm and are primarily found in the tidemark region, where they have been linked to elevated alkaline phosphatase activity [80]. Along with pathological crystals of calcium, they also have several modified protein contents and lower proteoglycan content. Additionally, synoviocytes’ markedly increase IL-1β production, cyclooxygenase (COX)-2, and tumour necrosis factor (TNF)-α suggesting that EVs play a role in the inflammatory process that occurs with OA. In fact, synoviocytes and chondrocytes both release EVs into the synovial fluid (SF) of OA patients [81]. Chondrocytes easily endocytose EVs derived from OA SF, and EV cargo analysis has revealed an upregulation of miR-200c in EVs derived from OA SF. Therefore, EVs containing miR-200c may aid in the maintenance of cartilage homeostasis by suppressing inflammation and boosting chondrocyte anabolic activity [74]. SF in rheumatoid arthritis (RA) has a relatively large number of EVs, such as microvesicles (MVs) and exosomes, and this is directly related to the progression of the disease. MVs in SF are derived from granulocytes and monocytes, as well as from lymphocytes to a lesser extent. MVs promote the production of thrombin, which contributes in local hypercoagulation in RA patients’ inflamed joints [82,83]. The appearance of citrullinated proteins, which are recognized as RA autoantigens, were found in exosomes derived from SF in conjunction with IgG and IgM, indicating they might be responsible for the induction of RA [84]. Platelet-derived MVs are rarely found in SF but are abundant in the plasma of those who are suffering from rheumatoid arthritis. EVs generating SF cells, specifically granulocytes and monocytes, intercellular adhesion molecule (ICAM), and VEGF (vascular endothelial growth factor) were found to generate MVs that cause synovial fibroblasts to secrete a different set of cytokines by increasing the expression of proteins such as MCP-1 (monocyte chemoattractant protein), RANTES (also recognized as chemokine (C-C motif) ligand 5), and IL-6 and IL-8, 1, all of which play a role in inflammation [83]. Synovial fibroblast-derived exosomes in rheumatoid arthritis contain tumour necrosis factor (TNF)-α in a membrane-bound form that is lacking in the exosomes of OA patients [85].

### 5.1. Isolation of Extracellular Vesicles

**Figure 5:**
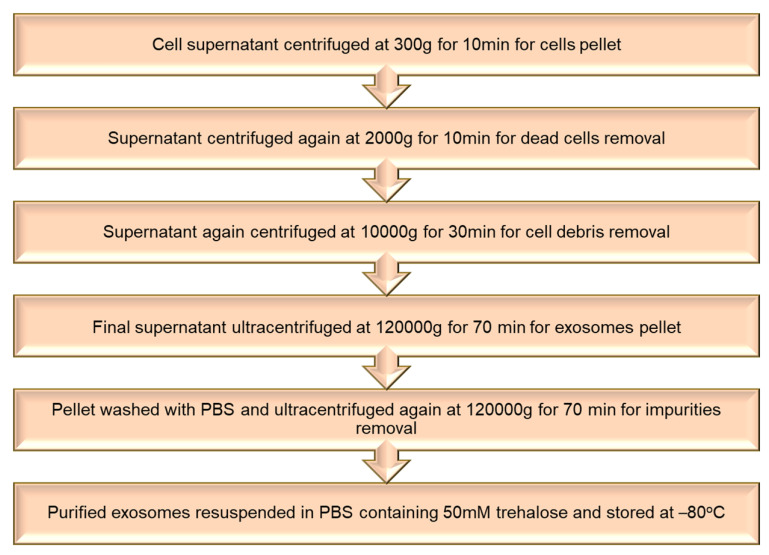
Isolation of exosomes.

Exosomes were harvested by seeding cells 2.0 × 10^6^ per well and allowing them to grow 70–80% confluent. Then these cells were incubated in a serum-free medium for 24 h. Exosomes were extracted from the conditioned medium of the cells by different cycles of ultracentrifugation at 4 °C, as mentioned in the flowchart (Figure 5). The pelleted exosomes were washed with PBS having 50 mM trehalose and stored at −80 °C for further usage. The Bradford assay was used to calculate the number of exosomes [86].

### 5.2. Loading of Therapeutic Agents in Extracellular Vesicles

Various researchers have found that EVs isolated from numerous cell types can have a curative effect in treating arthritis. In 2005, researchers began looking into the effects of dendritic cell (DC)-derived exosomes for arthritis treatment. They demonstrated that the systemic administration of DC-derived exosomes treated with IL-10 could prevent the development of arthritis induced with murine collagen and lessen the severity of pre-existing arthritis. These findings imply that exosomes involved in the inhibition of autoimmune and inflammatory responses can be secreted by immature DCs. As a result, exosomes produced by DC could be a brand new, cell-free therapy for autoimmune diseases [87]. Zhang et al., 2018, investigated the cellular progressions influenced by MSC exosomes as well as the mode of action governing exosome-facilitated responses in cartilage repair. They showed that MSC exosomes have the potential to repair and revitalize osteochondral defects of critical size through an organized, multifaceted response that includes increasing migration, proliferation, and synthesis of the matrix, decreasing apoptosis, and modifying immune reactivity. These findings suggest that MSC treatment is highly effective for treating complicated tissue injuries such as joint injuries and possibly osteoarthritis [88]. Various biomimetic nanomedicines are listed in Table 2.

Using an immunocompetent model of rat, researchers analysed the effects of MSC-derived exosomes on the inflammatory process, condylar cartilage, nociceptive behaviour, and subchondral bone healing for the treatment of temporomandibular joint osteoarthritis (TMJ-OA). Based on the observations, MSC exosomes appear to enhance TMJ healing and rejuvenation in OA by coordinating several cellular processes aimed at re-establishing matrix and joint homeostasis. This research proves that a cell-free exosome-based therapy has translational potential for alleviating TMJ pain and degeneration [94].

Recently, one research group created a biomimetic exosome (Exo) encasing a dexamethasone sodium phosphate (Dex) nanoparticle (Exo/Dex) for whom the outer layer was altered with folic acid (FA)–polyethylene glycol (PEG)–cholesterol (Chol) to attain active targeting of FPC-Exo/Dex. Due to PEG-Chol-FA alteration, these particles exhibit the benefits of other formulations in addition to a higher stability and relatively long persistence. These modified exosomes guarded bone and cartilage effectively in CIA mice and were endocytosed more effectively in vitro than free Dex or Exo/Dex. These results recommend that the FPC-Exo/Dex system developed could be an efficient drug delivery system for glucocorticoids to use in the treatment of RA [86].

**Figure 6:**
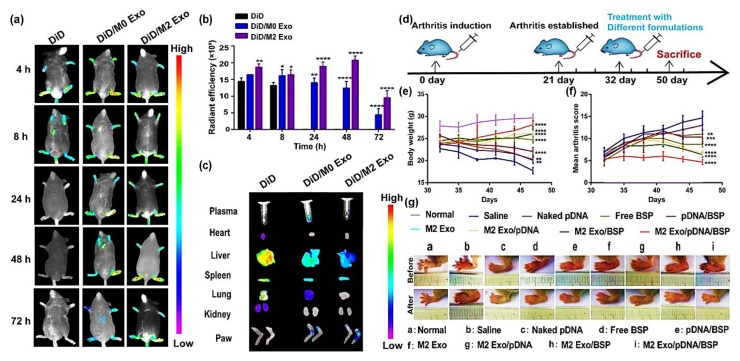
DiD-labelled formulation in vivo biodistribution. (**a**) Mice imaging with CIA at various time points after IV injection with DiD/M2 Exo, DiD/M0 Exo, free DiD (n = 3). (**b**) Ankle joint analysis of fluorescence intensity. * *p* < 0.05, ** *p* < 0.01, **** *p* < 0.0001. (**c**) DiD labelled and free DiD formulations’ biodistribution in blood as well as different organs after 72 h of injection. (**d**) Quantitative evaluation of M2 Exo/pDNA/BSP therapeutic efficacy in CIA mice showing the establishment of RA and treatment procedure. Measurement of (**e**) body weight and (**f**) average articular score. *** *p* < 0.001, **** *p* < 0.0001.(**g**) Images of mice hind legs both before and after treatment with numerous formulations of drugs. Adapted from [92] with permission.

You et al., 2021, developed exosomes derived from metabolically engineered stem cells to regulate macrophage heterogeneity in the treatment of RA. These exosomes could accumulate efficiently in the inflamed joints after being administered systemically to CIA mice, triggering an anti-inflammatory events cascade via regulation of the macrophage phenotype. Exosomes that have been modified in this way were 10 times more therapeutically effective than the unmodified exosomes [95]. M2-type-macrophage-derived biomimetic vector exosomes (M2 Exo) were used to encapsulate plasmid DNA that encodes interleukin-10 (IL-10 pDNA), an anti-inflammatory cytokine and betamethasone sodium phosphate (BSP), a chemotherapeutic drug. The synergism of BSP and IL-10 pDNA enhanced M1-to-M2 macrophage polarisation by lowering the pro-inflammatory cytokines’ (IL-1, TNF-α) secretion and elevating the IL-10 cytokine expression, which was responsible for the in vitro efficacy of this co-delivery system. M2 Exo/pDNA/BSP had a robust therapeutic effect in an RA mouse model, with more significant anti-inflammatory activity and efficient deposition at inflamed joint sites (Figure 6). This system’s potential for M1-to-M2 macrophage re-polarization-based RA therapy is encouraging [92].

Recently, some researchers utilized microvesicle-based biomimetics for the treatment of arthritis. Wang et al., 2022, prepared pH-sensitive biomimetic-based drug delivery carriers of microvesicles derived from macrophage (MV)-coated nanoparticles of zeolitic imidazolate framework-8 encapsulating methotrexate (MV/MTX@ZIF-8) for targeted RA treatment. 1,2-Distearoyl-sn-glycero-3-phosphoethanolamine-N-[folate(polyethylene glycol)-2000] was added further to MV/MTX@ZIF-8 to form FPD/MV/MTX@ZIF-8 that showed enhanced active targeting as well as immune evasion with extended retention. The developed nanoparticles had potent anti-inflammatory as well as cartilage and bone protective properties in CIA rats. Along with that, it also showed high drug loading efficiency (DLE), the ability to target inflammation, and pH-responsive drug release [90]. They also produced coated ZIF-8 nanoparticles with microvesicles derived from macrophages for sustained, localized dexamethasone delivery to arthritic joints. Experimental evidence from both in vitro and in vivo settings established that the nanosystem allowed for prolonged drug release in inflammatory joint tissues due to its excellent drug encapsulation and loading efficiency, good stability, and longer circulation time. These findings have implications for the creation of novel, more efficient nanoplatforms by providing new insights for developing camouflaged drug carriers to avoid phagocytosis and lengthen their circulation time [96].

## 6. Platelets Biomimetics System for Arthritis

Platelets have long been appreciated for their roles in haemostasis, but it is now widely accepted that they are also crucial in inflammatory and immune responses [97]. Platelet activation produces numerous inflammatory mediators, including chemokines, cytokines, and growth factors, which amplify inflammatory reactions, enhance angiogenesis, and recruit various cells in arthritis lesions [98]. Platelets can regulate a wide variety of immune cells because of their ability to secrete inflammatory molecules and express immune-related receptors [4]. Blood vessel damage is an inevitable consequence of inflammation, so platelets are called in to provide haemostasis in these areas. As a result, platelet-membrane-coated nanoparticles may be furnished with a platelet homing capability for inflammation sites [19]. Because of their many different roles in the body, platelets (PLs) have served as a source of motivation for the creation of motors that are enclosed in a cell membrane. PL membranes have now been isolated and encapsulated onto synthesized nanomedicines for imitating properties of platelets such as selective adhesion to ruptured vasculature and tumours, lower cellular uptake by immune cells, and increased binding to platelet-adhering pathogens [99].

Additionally, platelets are promising candidates for targeted drug delivery strategies because of their particular affinities for immune tissues and cells in RA lesions. Platelets may be removed from blood and re-transfused after being modified ex vivo, or hitch a ride with nanomedicine and prodrugs to obtain optimum drug delivery [100]. Additionally, platelet membrane cloaking techniques and artificial platelet synthesis have been made possible by the development of nanotechnology, resulting in modalities that mimic the properties of platelets. The functional and morphological modifications that occur during platelet activation can also cause drug release [4]. Nanoparticles coated with platelet membranes have received much attention over the previous few decades because of the ability of the platelets to evade the immune system, adhere to subendothelial cells, and interact with pathogens [101]. The camouflage technique of platelets is primarily based on the protein profile on their surface, which permits natural physio-pathological affinity and inflammation site targeting [102].

**Figure 7:**
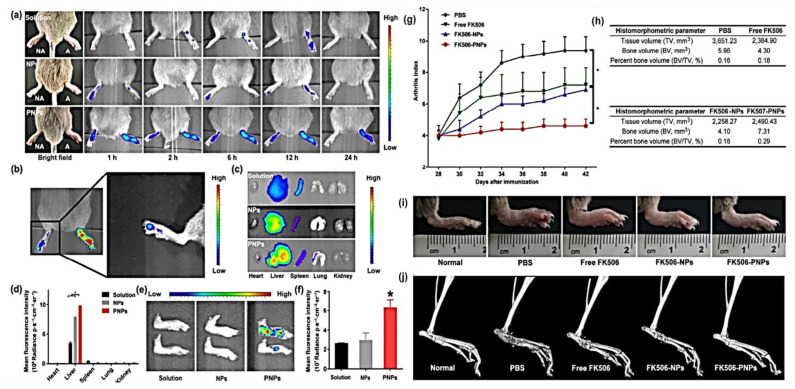
PNPs in vivo and ex vivo imaging in CIA mice. (**a**) NIRF imaging in vivo of arthritic (right) and non-arthritic (left) paws from CIA mice (n = 3) at various times following intravenous injection. (**b**) Inflamed right paw fluorescence images with only one arthritic toe. (**c**) NIRF imaging ex vivo of major organs after 24 h of nanoparticle injection. (**d**) Major organs mean NIRF intensity (n = 3; mean ± SD), *p* < 0.05 compared to a solution or the NP group. (**e**) Twenty-four-hour post-injection inflamed paws ex vivo NIRF image from the NP as well as PNP groups. (**f**) Paw inflammation intensity as measured by mean NIRF (n = 3; mean SD), * *p* < 0.05 vs. solution and NP groups. (**g**) CIA mice therapeutic efficacy. Average rheumatoid arthritis index as a function of the time since the first immunization, * *p* < 0.05. (**h**) Micro-CT data of hind paw quantitative analysis. (**i**) Hind paw representative pictures from CIA mice in various treatment groups as well as normal mice that did not receive any treatment. (**j**) Mice with and without CIA were studied using micro-CT scans of their hind paws, and the results were reconstructed in three dimensions. Adapted from [103] with permission under license CC-BY.

Depending on the platelets’ biological function, He et al., 2018, first engineered platelet-membrane-coated PLGA nanoparticles for targeted drug delivery in rheumatoid arthritis. They made the platelet-mimetic NPs by coating PLGA-FK506 with platelets (PNPs). Membrane proteins of platelets (glycoprotein VI and P selectin) were shown to interact with RA synovial tissue markers (CD44 and collagen IV), proving that PNPs have a particular affinity for synovial tissue, which is inflamed. The anti-inflammatory effects of FK506-PNPs were more significant in the CIA mouse model compared to those of FK506 alone and FK506-PLGA NPs (Figure 7). This research unveils a novel biomimetic-based targeting strategy with promising applications in RA therapy [103].

### Isolation of Platelet and Membrane Derivation

**Figure 8:**
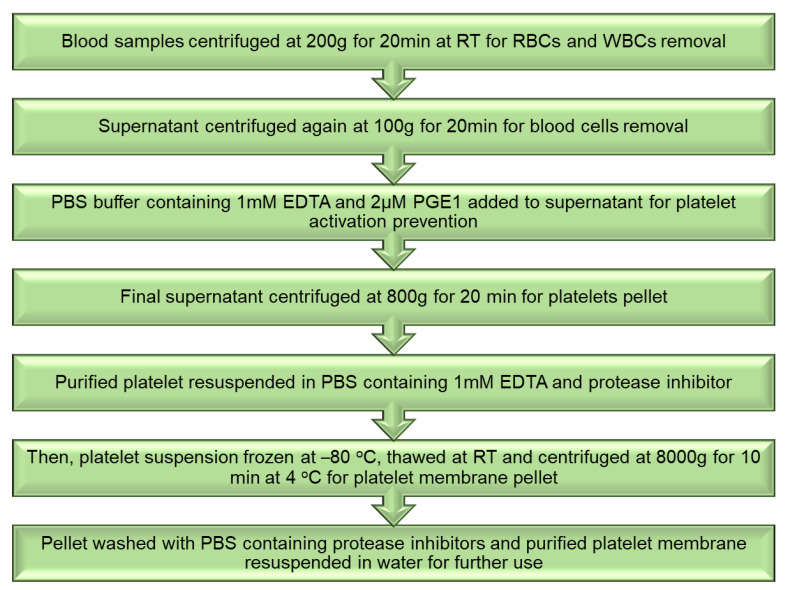
Isolation of platelet and membrane derivation.

Whole blood was used to separate the platelets by utilizing differential centrifugation. Rats had their abdominal aortas punctured, and their blood was collected in a tube with ethylenediaminetetraacetic acid (EDTA) as an anticoagulant. Platelet isolation was performed at room temperature, while membrane derivation was performed at 4 °C (Figure 8). The preparation of the platelet membrane was purified and then resuspended in water for future use [103,104].

## 7. Functionalization of Biomimetic Nanomedicines

The primary issue with standard nanoparticle administration is that it exhibits toxicity and is quickly eliminated by the immune system. Target specificity, biocompatibility, and high stability are aided by the functionalization of biomimetic nanomedicines by biomolecules and components obtained from cell membranes [105]. Because of the ability of the lipidic cell membrane of the biomimetic nanoparticles to bind with an antibody and peptide, functionalization not only prolongs the circulation of the biomimetic nanoparticles within the body and reduces opsonization but also aids in chemotaxis movement, adhesion function, and extravasation, allowing for active targeting towards a particular area in the body [106].

The coating of biomolecules that forms on the surface of nanoparticles when they are distributed in biological fluids aids in the adsorption of certain proteins that help in active targeting [107]. The use of biomimetic nanomaterials has enabled the association of complex structures that are linked to various cell membrane structures and that are impossible to replicate with synthetic materials [108]. For the biofunctionalization of the nanoparticles, different cell sources can be used, such as different lymphocytes, platelets, red blood cells, bacterial cell membranes, cancer cell membranes, and mesenchymal stem cells, as each of these offers a unique advantage for customizing nanoparticles based on their characteristics [102,109]. Due to its abundance in the body, the lack of cellular organelles such as the nucleus, and lengthy circulation period, the erythrocyte cell membrane continues to be the most practical material in the synthesis of biomimetic nanoparticles [110]. It also has the benefit of evading the immune response since it has numerous proteins on its surface, notably CD47 [111].

In most cases, biomimetic nanomaterials are created using a quick and easy top-down production method [112]. Electrostatic attraction or physical extrusion between the core material of the nanoparticles and the cell membrane vesicles produces biomimetic nanoparticles [64]. A self-assembly process, nanoprecipitation, or solvent evaporation from a double emulsion is used to create the core material [104], while hypotonic lysis is used to create the cell membrane vesicle [113].

With the use of this technology, a number of illnesses may be targeted. For instance, a biofunctionalized PDT nanovector was created in conjunction with targeting ligands to boost localization at the tumour location [114]. By utilizing an enzyme-responsive RBC membrane biomimetic nanoparticle, vancomycin was administered to the body [115]. Another study added folate to the biomimetic nanoparticle and AS1411, a nucleolin-targeting aptamer, to improve active targeting [116]. Numerous immune system cells, including monocytes, neutrophils, lymphocytes, basophils, and eosinophils, are found inside the lesions of rheumatoid arthritis, which cause inflammation. The cell membranes of these immune system cells will therefore aid in the effective active targeting of biomimetic nanoparticles. In the research, a macrophage-derived vesicle was coated with tacrolimus-coated nanoparticles since macrophages are the main cells that infiltrate the locations of arthritic lesions [4]. By inhibiting osteoclastogenesis, this technique not only assists in attaining active targeting but also aids in competitive binding with the inflammatory cytokines, preventing bone degradation by arthritis [69]. Dexamethasone sodium phosphate, which is used to treat rheumatoid arthritis, is recognized to have a short half-life and several side effects; therefore, to get around these problems, macrophage-derived microvesicles were created in which a metal–organic framework was formed by encapsulating the medication onto the zeolite imidazole framework-8, and thus the MV/Dex/ZIF-8 biomimetic drug delivery system was formulated [96].

### 7.1. Biomimetic Nanomedicines with Targeting Ligand for Arthritis

**Figure 9:**
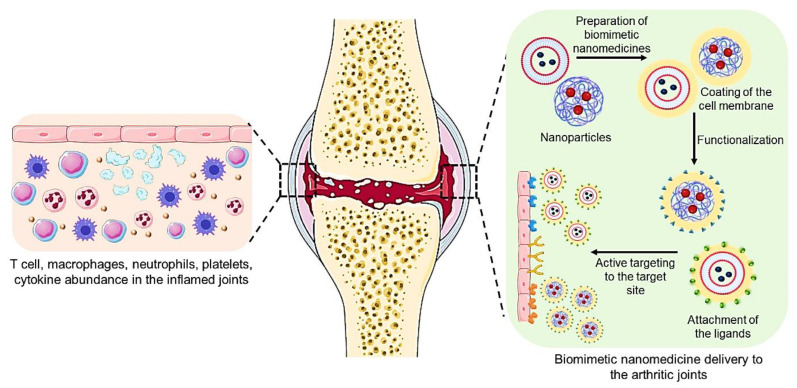
Biomimetic nanomedicines with targeting ligands.

By combining biomimetic nanoparticles with ligands that have an affinity to connect with receptors found on the surface of target cells, active targeting of the nanoparticles is made feasible. Utilizing ligands made up of aptamers, antibodies, small molecules, and peptides allows for the targeted delivery of medications through these biomimetic nanoparticles [117]. The functionalization of biomimetic nanomedicines with targeting ligands is picturized in Figure 9.

The biomolecular cell membranes may effectively conceal the core of the nanoparticle, allowing the medicine to be actively targeted at the lesions of rheumatoid arthritis. Neutrophils were used in research to coat the nanoparticles, which helped them become more selective for use on inflamed cells. Due to LFA-1’s presence on the neutrophil membrane and its association with ICAM-1, which is expressed on chondrocytes, precise targeting was made achievable [19]. Due to platelet activation, several inflammatory mediators, including growth factors, chemokines, and cytokines, are drawn to the lesions of arthritis [4]. Platelet-shrouded nanoparticles are a fascinating alternative cell type that might be employed to create biomimetic nanoparticles. He et al. created a platelet-membrane-masked PLGA nanoparticle to administer the immunosuppressive medication FK506 [103]. The CD44 and collagen IV markers found in synovial tissue interact with the glycoprotein IV and P selectin membrane proteins on platelet membranes; this connection also contributes to the efficacy of the medication-targeting mechanisms [118]. Since exosomes are known to control osteoclastogenesis and the immune response, they are another kind of biomolecule that may be used to create biomimetic nanoparticles for the treatment of arthritis [119]. They have a better capacity to permeate the tissue than any other cell membrane camouflage mechanisms, making them more favourable. Dexamethasone sodium phosphate was coated with exosomes from macrophages to create biomimetic nanoparticles, which were subsequently FA-PEG-Chol surface modified. This approach demonstrated increased inflammatory capability when compared to previous liposomal vectors [65]. Since SPARC (secreted protein acidic and rich in cysteine) is overexpressed in the synovium and synovial fluid, methotrexate-loaded human serum albumin nanoparticles were created to target it. In addition, because arthritic joints use more albumin than healthy joints, albumin can be specifically targeted to these joints [93].

Human serum albumin (HSA) is employed as an agent to create a biomimetic nanoparticle system because proteins such as this have a tendency to concentrate in the synovial tissue of patients with arthritis. The synovial tissues and fluids contain significant levels of osteonectin, also known as SPARC (secreted protein acidic and rich in cysteine), with a strong affinity for HSA. On the basis of this, a biomimetic system containing HSA for the drug methotrexate was created to treat arthritis [93]. By adding mannose to the biomimetic system, Lyu et al. further modified the HSA to administer neutrophil-specific methotrexate to RA lesions. When the mannose-coated biomimetic system was compared to the non-coated system, it was discovered that it was distinguished by low cytokine release and that the rate of bone degradation and joint oedema was decreased [120].

Due to their covalent connections, biomimetic nanoparticles can be functionalized with the help of DNA molecules [121]. Using aptamers, microRNA, and DNA decoys to deliver the gene increases immunological compatibility, increases the stability of the nucleic acid, and reduces its degradation [122]. Folate-coated chitosan DNA nanoparticles and an IL-1 receptor antagonist gene were used to target IL-1 in arthritic patients [123]. Since hyaluronic acid is not only biocompatible by nature but also has a propensity to attach to a number of indicators linked to the aetiology of arthritis, including CD44, LYVE-1 receptor, and proteins such as hyaladherins, it may be employed as a targeting molecule and an appropriate drug carrier [124]. As a result, HA-based nanoparticles may easily target macrophages that express the CD44 receptor [125]. It was discovered that because the biotinylated-SLeX ligand has an affinity for selectin, attaching it to PLGA microspheres enables active targeting towards inflammatory tissue [126].

Due to the fact that P selectin, ICAM-1, VCAM-1, and E-selectin are among the inflammatory indicators that are overexpressed during the inflammation phase, PLGA nanoparticles were created by biotinylating different antibodies against these markers [19].

Due to their ability to release cytokines, activate chemokines, and attract other inflammatory cells, M1 macrophages are one of the most often recruited inflammatory cells in rheumatoid arthritis. They are also in charge of degrading cartilage and removing the surface of the bones.

Considering this knowledge, Shi et al. created a biomimetic formulation using an umbilical vein endothelial cell membrane. This formulation was then coated with the drug hydroxychloroquine nanoparticles and functionalized by TRAIL (tumour necrosis factor-related apoptosis-inducing ligand), a ligand that targets the areas of inflammation. The created nanoparticles can cause M1 macrophages to undergo apoptosis while also delivering an anti-rheumatic drug to the affected tissue [68]. Folic acid, hyaluronic acid, and dextran sulfate are typically utilized to target macrophages since inflammatory conditions express the folic acid receptor, CD44 receptor, and scavenger receptor [127].

Numerous targeting peptides, such as ART-1 and RGD, as well as compounds, such as quinolones, can be employed to target the endothelial cells to increase the targeting effectiveness of the nanomedicine toward the inflamed tissue [128]. A platelet biomimetic nanoparticle was designed based on the theory that P selectin mediates the contact between inflammatory tissues and platelets. This interaction results in a high concentration in the inflamed tissue and prolonged circulation time. When functionalized with FK506, this bioimitated nanoparticle significantly reduced joint inflammation [103].

### 7.2. Stimuli-Responsive Biomimetic Nanomedicine

Due to inadequate tissue accumulation and many off-target consequences, the response rate to different treatment approaches for arthritis remains low. As a result, much research is being conducted to improve arthritis treatment utilizing nanotechnology and targeted drug delivery. Various biomimetic therapeutic options are being used to increase tissue penetration, provide specificity, have longer circulation times, and enhance retention, including ligand-based active directing and stimuli-responsive active targeting. Active therapy uses a variety of stimuli, including pH responsiveness, magnetic agents, photothermal stimuli, redox stimuli, and ultrasonic stimuli [4].

**Figure 10:**
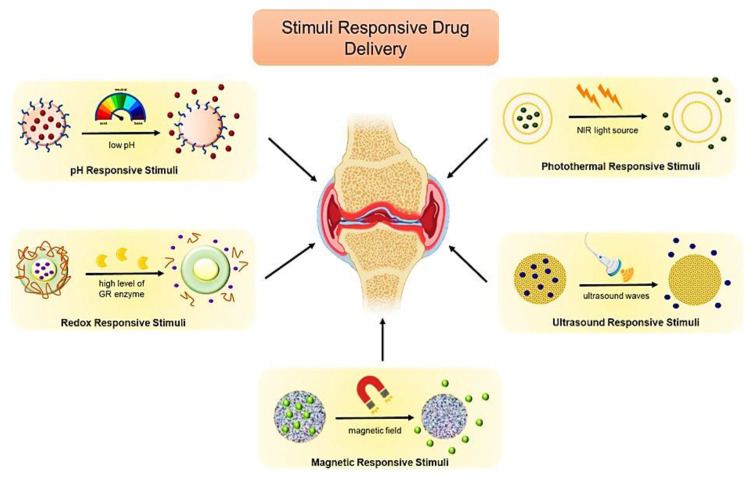
Stimuli-responsive biomimetic nanomedicines.

The body has varied pH gradients depending on the level of the organ or the different pathological conditions, and these variations can be used to actively transport medications to a specific area of the body [129]. Different approaches have been tried to deliver therapeutic agents to the tumour’s microenvironment, and because the tumour has an acidic environment, labile acid polymers can be utilized to ensure that the drug is delivered just where it is needed. For instance, when coated with the RBC membrane, dextran nanoparticles carrying the drugs Lexiscan and doxorubicin displayed pH sensitivity to the acidic endosome environment [130]. Various stimuli-responsive biomimetic nanomedicines are shown in Figure 10.

Inflammation is a crucial factor in the development of arthritis, and it is connected to a fall in pH levels. For instance, the normal physiological pH value is 7.3, but the inflammatory tissues of arthritis have a pH of roughly 6.6. As a result, pH-sensitive stimuli can be employed for the successful treatment of arthritis. Due to endothelial cell discontinuity brought on by increased angiogenesis and high vascular permeability, nanoparticles can quickly enter arthritic areas. When these nanoparticles reach the site of action, the low pH levels cause the drug to release [131]. Methotrexate, an anti-rheumatic drug, was transformed into a nano-drug system encapsulated by the acetylated dextran polymer, giving it the property of being pH sensitive to lower toxicity and boost its efficacy [132].

In a different study, a pH-sensitive biomimetic nanosystem was developed using a membrane vesicle made of macrophages that contained the medication methotrexate and were covered with a zeolitic imidazolate framework. To specifically target the folic acid on the surface of macrophages, they were further treated by 1,2-distearoyl-sn-glycero-3-phosphoethanolamine-N-[folate (polyethylene glycol)-2000]. The study indicated that the system had good entrapment efficiency and that the therapeutic agent was released effectively at the acidic pH. The system demonstrated adequate safety and effectiveness, good active targeting, and the capacity to circulate over prolonged periods [90].

The variations between the intracellular and extracellular components can be examined to enable active targeting of the drug molecules. For example, glutathione has a lower concentration in the extracellular compartment, while its intracellular concentration is substantially higher [133]. This characteristic can be used for active targeting; for example, human liver cancer cells were used to coat disulphide bridged mesoporous particles in a biomimetic drug delivery system for the drug berberine. Disulphide bonds were broken, and the drug was actively released in the GSH presence in the tumour cells. Additionally, the coated cell membrane reduced the drug’s blood clearance and increased the accumulation of the nanosystem in the cancer tissue [134]. Since the reducing and oxidizing spaces would have distinct redox potentials, the property of the elevated ROS level can be used for medication administration in inflammatory circumstances. ROS is mainly created during oxidative phosphorylation in chronic arthritis due to the oxidative burst produced by the active phagocytes [135]. By coating the nanoparticles with an amphiphilic polymer containing glutathione, which was eventually acted on by the GR enzyme present in the inflamed joints, Lima et al. were able to offer controlled drug release of the drug dexamethasone [136].

The magnetic-containing nanoparticles can be guided to the target region of action using an external magnetic field. Additionally, this can facilitate the nanoparticles’ passage across several physiological barriers [137]. Iron oxide nanoparticles have been used for various applications, including tissue regeneration, imaging, drug delivery, and cell targeting. The RBC biomimetic membrane encapsulated these nanoparticles, enhancing their circulation time and significantly slowing their blood removal [111]. Another instance of biomimetic action was when a magnetic nanowire covered with platelet membranes was used to detoxify the body by removing pathogens and poisons [138]. A detoxification program employing ultrasound and a nanosystem with cell membrane coating helped remove the poisons that caused pores to develop. The nanosystem’s motors, composed of citrate-stabilized gold nanowires, were protected by the cell membrane coating, which also served as a barrier against biofouling [139]. Using the system of nanobombs created by Zhu et al. resulted in a decrease in the inflammation of the synovium and damage to the cartilage. He coated the centre of the perfluorocarbon with FA-modified PEG phospholipid and used ultrasonic stimulation to provide the targeted medication release [140].

Two new stimuli-based treatments, PDT (photodynamic therapy) and PTT (photothermal therapy), are frequently utilized to target medication distribution. The use of photosensitizers for PDT treatment is still restricted due to their low absorption and restricted site specificity. By covering the photosensitizer-based nanoparticles with the cell membrane imitating system, this may be readily avoided [141]. Similar to this, PTT biomimicking platforms have emerged in which light-sensitive compounds are enclosed within the cell membrane. Consequently, the medicine may be readily delivered to the target region by externally applying the light stimulus. For instance, the cancer cell membrane could enclose the medication doxorubicin and the photothermal agent indocyanine green [142]. NIR or external heat stimuli can be used to trigger the thermoresponsive materials’ thermal reactions to treat arthritis [135].

For instance, the release of methotrexate from PLGA nanoparticles at the inflammatory site was enhanced by the heat produced by the gold nanoparticle when exposed to infrared light [143]. Schisanlactone E, an anti-inflammatory medication, was enclosed in Prussian blue nanoparticles and then coated with a hybrid RBC-RAFLS system membrane to create multifunctional nanoparticles. This technology was used in conjunction with the photothermal effect, demonstrating that controlled drug release was feasible. Additionally, there were improved clinical results, high therapeutic effectiveness, and target drug accumulation at the sites of arthritic inflammation [66].

Multistimuli-responsive drug delivery refers to using more than one stimulus to target different liposomes, dendrimers, conjugates, and micelles in the drug delivery system. To treat arthritis, Kim et al. created PLGA nanoparticles that were loaded with the medication methotrexate. These nanoparticles not only offered chemotherapeutic therapy but also aided in the imaging of arthritis owing to the employment of an external magnetic field and NIR radiation [144]. Hu et al. used Prussian blue nanoparticles in conjunction with indomethacin to treat arthritis. The hyaluronic-acid-modified nanoparticles were biomimetic in nature and helped the medicine target inflammatory arthritis, make it more soluble, and evade the immune system. Due to pH-driven nanomedicine and subsequent integration of the photothermal effect, the technology was extremely favourable in reducing the inflammatory impact and having low side effects [145].

## 8. Challenges for Biomimetic Nanomedicines

Researchers are still dealing with many difficulties in the expansion of biomimetic nanomedicines. The integrity of the cell membrane is the central area of concern since the function of the cell membrane determines cell integrity. The treatment of the cell membrane with a hypotonic solution or a lysis solution during the camouflaging method is likely to affect the protein sequence and structure of the cell membrane, which is a feature of the membrane integrity. Consequently, part of the cell membrane fragments is lost during extrusion and fusing. Researchers find it difficult to characterize this loss, so advanced methodologies are required to determine the integrity. The additional concerns linked to this include the heterogenicity issues brought on by various cell membrane types, the reproducibility and complexity issues, and the chemical and physical stability of the cell membrane [102].

The correct orientation assembly between the cell membrane and core nanoparticles and the extraction of the cell membrane are necessary for the successful development of biomimetic nanomedicines. This will ensure that all the necessary proteins of the cell membranes are exposed and arranged correctly to perform their biological activity [146]. The extraction of cell membranes frequently involves the use of differential gradient or density gradient centrifugation. It might be challenging to determine the proper centrifugation and cell fragmentation parameters. The concern allied with the integrity of the cell membrane is that once the membranes are retrieved and separated, they are known to degrade quickly in response to a lack of nutrition and cytoplasmic support [147]. The development procedures should be kept straightforward since adding complexity to the processing of the cell membrane raises the likelihood that it will not successfully mimic a biological system. Furthermore, it has been discovered that it is difficult to position the cell membrane so that all protein molecules on its surface are exposed correctly. For instance, a study found that only 84 percent of the RBC cell membranes are oriented in the correct position when they are used in a biomimetic system [148].

Even though the usage of biomimetic nanomedicines in research has significantly risen due to the complexity of the systems involved, no solo system has yet been clinically translated. There is a need of clinical translation for biomimetic nanomedicines to ensure the safety and efficacy of the system. The variety in the size, shape, physicochemical qualities, and various receptors and proteins present on the cell surface pose researchers the most significant problems in terms of stability [149]. The fragile nature of the membrane proteins makes them highly vulnerable to denaturation during production and storage [150]. Since lab-scale manufacturing is unsuitable for large-scale production, the clinical translation of this method has proven challenging. Therefore, an industrially scaled approach that is efficient, reliable, and repeatable is needed to extract the cell membrane and effectively coat the nanoparticles. When there is batch variability, contaminants are present, the coating is insufficient, or the coating efficiency is low; hence, caution should be used [151]. When the immune system responds negatively, it can have harmful effects, and using cancer or bacterial cell membranes to create a biomimetic nanosystem can cause this negative reaction. Normal cell membranes should also be used with caution since there may be some discrepancies in the final cell membrane when it has been removed using different techniques and procedures from the native cell membrane [152].

The main hurdle is to thoroughly investigate the clinical translation of biomimetic nanosystems so that the necessary regulatory guidelines can be created to establish the evaluation criteria for the safety requirements, protein expression, quantification of the coating, stability, and target specificity of the nanoparticles [150]. A quality-by-design (QbD) methodology is applied to create and design the formulation of the biomimetic nanoformulation and maximize its scalability and effectiveness. Through its data-driven procedure, the QbD technique results in fewer experimental runs and a higher understanding of the processes [153]. The surface charge, surface proteins, particle size, membrane orientation and integrity, protein corona formation, cellular selectivity, possible immunogenicity, evaluation of safety, and loading efficiency are some of the important quality features for biomimetic nanosystems [154].

The preparation of biomimetic drug delivery carriers is primarily focused on increasing the biocompatibility of the existing delivery systems and effectively directing them to the intended location. The nanoparticles should be generated in such a way that they do not interfere with the structural functions of the cell membrane or its ability to express proteins. The capacity of the core nanoparticles to sustain the cell membrane is an important aspect [146]. To conquer the challenges of the biomimetic nanomedicines, researchers should find an alternate method for the isolation of the cellular membrane that will cause less membrane damage. In the future, efforts should be taken to reduce the immunologic responses for biomimetic nanomedicines.

## 9. Conclusions

Biomimetic-based therapeutic systems are astonishing carrier systems for targeted drug delivery in various diseases to minimize the off-target effects. This review discussed several cellular-component-based biomimetics systems and their functionalization, the modification of biomimetics systems for targeting, and their efficiency in arthritis treatment. The biomimicking nature of these cellular-system-based biomimetic nanomedicines has conquered various limitations of traditional drug delivery systems. These biomimetic nanomedicines exhibit lower tissue toxicity due to targeting and enhanced circulation time, and are capable of crossing biological barriers that will further enhance the therapeutic effect. The integrity of cellular components should be maintained when composing the biomimetic systems. In the translational stage, various properties such as shape, size, various proteins, or receptors on cellular components lead to a struggle to maintain integrity and stability. Even though biomimetic systems have shown tremendous efficiency in preclinical studies, much research needs to be carried out for the successful clinical translation of these biomimetic systems.

## Figures and Tables

**Table 1:** Various therapeutic targets and their agents for arthritis treatment.

Targets	Agents	Phases
**Cytokine**		
TNF	Adalimumab	Marketed
	Infliximab	Marketed
	Etanercept	Marketed
	Certolizumab	Marketed
	Golimumab	Marketed
IL-1R	Anakinra	Marketed
IL-1	Canakinumab	Marketed
	Gevokizumab	Marketed
	Rilonacept	Terminated
IL-6R	Tocilizumab	Marketed
IL-6^a^	Sarilumab	Marketed
	Clazakizumab	Marketed
	Olokizumab	Marketed
	Sirukumab	Marketed
IL-2	MEDI5117	Terminated
IL-10	Dekavil	Phase 1
IL-15	AMG-714	Phase 2
IL-18	rhIL-18BP	Phase 1
IL-17	Secukinumab	Phase 3
	Ixekizumab	Phase 2
IL-17R	Brodalumab	Terminated
IFN-γ	Fontolizumab	Terminated
**Chemokines**		
CCL2	p8A MCP-1	Animal study
	ABN912	Phase 1
CCR9	CCX8037	Animal study
CX3CL1	E6011	Phase 1
CCR1	J–113863	Animal study
	BX147	Animal study
	BAY86-5047	Phase 2
	ZK811752	Phase 2
	CCX354	Phase 2
	BMS-817399	Phase 2
CCR2	MK-0812	Phase 2
	MC-21	Animal study
	MLN1202	Phase 2a
CCR5	SCH-X82	Phase 2
	Met-RANTES	Phase 2
	AZD5672	Phase 2
	Maraviroc	Terminated
	SCH351125	Phase 1b
CXCL10	MDX-1100	Phase 2
CXCL12	30D8	Animal study
CXCL13	mAb470	Animal study
CXCL16	IgG1 12-81	Animal study
CXCR1/2	Repertaxin	Animal study
	DF2162	Animal study
CXCR3	SCH546738	Animal study
	AMG487	Animal study
	JN-2	Animal study
CXCR4	Plerixafor	Animal study
	T140	Animal study
	AMD3100	Animal study
CXCR7	CCX733	Animal study
CCR7	8H3-16A12	Animal study
**Other Proteins**		
TLR4	NI-0101	Phase 2
GRK2	Paroxetine	Phase 2
MEK	ARRY-162	Phase 2
MMP-9	Andecaliximab	Phase 2
CD3	Otelixizumab	Phase 1
CD80	Abatacept	Marketed
BTK	ICP-022	Phase 1
	CC-292	Phase 2
	HM71224	Phase 1
	M2951	Phase 2
	GS-4059	Phase 1
IL-23	STA 5326 mesylate	Phase 2
	Guselkumab	Terminated
GM-CSF	Otilimab	Phase 3
	Gimsilumab	Phase 1
	Namilumab	Phase 2
	Mavrilimumab	Phase 2
	Lenzilumab	Terminated
JAK	Tofacitinib	Approved
	Baricitinib	Approved
	Filgotininb	Phase 3
	Upadacitinib	Approved
	Peficitinib	Phase 3
	Ruxolitinib	Phase 2
	Itacitinib	Phase 2
	Tasocitinib	Phase 2
	INCB018424	Phase 2
	VX-509	Phase 3
p38 MAPK	RO4402257	Phase 2
	PH-797804	Phase 2
	VX-702	Phase 2
	BMS-582949	Phase 2
	ARRY-371797	Phase 1
	SCIO-469	Phase 2
	SB-681323	Phase 2
IRAK-4	PF-06650833	Phase 2
	BAY1834845	Phase 1
	BAY1830839	Phase 1
	CA-4948	Phase 2
CD20	Rituximab	Phase 3
	Ocrelizumab	Terminated
	Ofatumumab	Phase 3
CD11a	Efalizumab	Phase 2
CD19	MDX-1342	Phase 1

**Table 2:** List of biomimetic nanomedicines for the treatment of arthritis.

Biomimetic System	Nanoformulation	Active Moiety	Size	Functionalization	Inference	Reference
Macrophage membrane vesicles	Prussian blue nanoparticles	siRNA	-	-	Photoacoustic-guided nanoparticles assisted better diagnosis and treatment	[89]
RBC-RAFLS hybrid membrane	Prussian blue nanoparticles	Schisanlactone	141.8 ± 10 nm	Hyaluronic acid	Synergistic chemo-/photothermal therapy with controlled and targeted release	[66]
Macrophage-derived microvesicle (MMV)	PLGA Nanoparticles	Tacrolimus	130 ± 14 nm		Morenhanced targeting than RBC-coated membrane	[69]
Macrophage-derived microvesicle (MMV)	Zeolitic imidazolate framework-8 nanoparticles	Methotrexate	147.7 ± 3.21 nm	1,2-distearoyl-sn-glycero-3-phosphoethanolamine-N-[folate (polyethylene glycol)-2000]	pH-sensitive release in acidic environment.	[90]
Neutrophil-membrane-coated	Pluronic F127 nanoparticles	Celastrol	51.25 ± 2.086 nm	R4F peptide	Macrophages targeted formulation with reduced hepatotoxicity	[91]
Exosome	Nanoparticles	IL-10 pDNA and betamethasone sodium phosphate	99.97 ± 4.77 nm	-	Combined therapy with synergistic effect	[92]
Extracellular vesicle (Exosome)	Nanoparticles	Dexamethasone sodium	128.43 ± 16.27 nm	Folic acid	Biocompatibility and no hepatotoxicity	[86]
SPARC (secreted protein acidic and rich in cysteine) in arthritis microenvironment	Albumin nanomedicine	Methotrexate	30.71 ± 4.62 nm	-	Longer retention and reduced systemic toxicity	[93]

## Data Availability

Not Applicable.

## Abbreviations

AB	Apoptotic bodies
BSP	Betamethasone sodium phosphate
COX	Cyclooxygenase
DC	Dendritic cell
Dex	Dexamethasone sodium phosphate
EDTA	Ethylenediaminetetraacetic acid
EVs	Extracellular vesicles
FA	Folic acid
HSA	Human serum albumin
ICAM	Intercellular adhesion molecule
MCP-1	Monocyte chemoattractant protein
MTX	Methotrexate
MVs	Microvesicles
OA	Osteoarthritis
PB	Prussian blue
PDT	Photodynamic therapy
PEG	Polyethylene glycol
PLs	Platelets
POX	Poly-2-oxazolines
PTT	Photothermal therapy
QbD	Quality-by-design
RA	Rheumatoid arthritis
RES	Reticuloendothelial system
SEC	Size-exclusion chromatography
SF	Synovial fluid
SPARC	Secreted protein acidic and rich in cysteine
TMJ-OA	Temporomandibular joint osteoarthritis
TNF	Tumour necrosis factor
TRAIL	Tumour necrosis factor-related apoptosis-inducing ligand
VEGF	Vascular endothelial growth factor
